# Analysis of influence factors of target polarization characteristics

**DOI:** 10.1038/s41598-023-49228-5

**Published:** 2023-12-08

**Authors:** Zhiwei Zhang, Zhiyong Yang, Gengpeng Li, Dong Chen, Xiaowei Wang

**Affiliations:** State Key Discipline Laboratory of Ordnance Launch Theory and Technology, Rocket Force Engineering University, Xi’an, 710025 China

**Keywords:** Optics and photonics, Physics

## Abstract

The target polarization characteristics reflect the important information about the medium material, surface texture, and structural characteristics of the target itself. In this paper, 9 kinds of samples with different materials, roughness, and surface texture direction are selected to measure the polarization characteristics in an outdoor environment, and the influencing factors of the target polarization characteristics are analyzed, where the influence of the surface texture direction on the spatial distribution of the target polarization characteristics is emphatically analyzed. The results show that the target polarization characteristics are affected by many factors such as material, roughness, surface texture direction, and detection position, and the greater the angle between the surface texture direction and the principal plane, the more concentrated the target polarization characteristics are in and around the principal plane, which could provide a theoretical basis for comprehensively mastering the target polarization characteristics, improving the ability of target polarization detection and recognition, and enhancing the confidence of the target polarization modeling and simulation.

## Introduction

As one of the important characteristics of light wave, polarization provides unique information different from intensity and spectrum, which contains rich information related to the inherent physical and chemical characteristics of the target, such as medium material^1^, surface texture^2^ and structural features^3^. Therefore, as a new optical detection method, polarization detection has been widely studied in recent years, especially in the field of military application^4,5^ target detection and recognition^6,7^ medical diagnosis^8,9^ and remote sensing^10,11^. Because the polarization characteristics reflect the intrinsic characteristics of the target material, the difference in the target polarization characteristics of different materials can provide important information for target identification and detection.

Researches on the polarization characteristics of typical targets have been carried out since the 1950s. Especially in the field of military application, relatively complete databases have been established for some typical military targets and backgrounds, and put into practical application. In 2000, the US Air Force Research Laboratory^12^ measured the polarization reflection characteristics of 12 different coating samples with the US federal standard coatings at 8 incident angles between the wavelength range of 0.9 μm and 1 μm, and the results showed that the degree of polarization was positive with the incident angle but negative with the reflectance. In the same year, Egan^13,14^ of New York University used a focal plane polarization camera to compare and analyze the polarization images of 9 positions on C-130 camouflage aircraft in 5 different sky backgrounds, and the results showed that the polarization reflection characteristics of natural background and camouflaged targets were quite different, and the camouflaged military targets could be well detected by polarization detection. Since February 2010, the U.S. Armament Research, Development, and Engineering Center (ARDEC) and the U.S. Army Research Laboratory^15^ have jointly carried out the spectral and polarimetric imagery collection experiment, using the hyperspectral, polarimetric, and broadband sensors to automatically collect the mid-wave and long-wave infrared imagery of 2S3 Self-Propelled Howitzer for 7 months at all hours, all-weather and from multiple angles. Sun et al. mainly focused on the polarization characteristics of snow^16,17^, leaf^18,19^, and soil^20,21^, and analyzed different influence factors.

Analysis of target characteristics is an important prerequisite for target detection and recognition, and a comprehensive understanding of the target polarization characteristics is conducive to improving the ability of target detection and recognition. In this paper, the polarization characteristics of 9 kinds of samples, including 3 brass plates with different roughness, 3 aluminum plates with different roughness, cardboard, plastic, and white paper are measured and analyzed in different detection positions by means of polarization imaging, so as to explore the influencing factors of the target polarization characteristics.

## Experiments

### Equipment and samples

**Figure 1:**
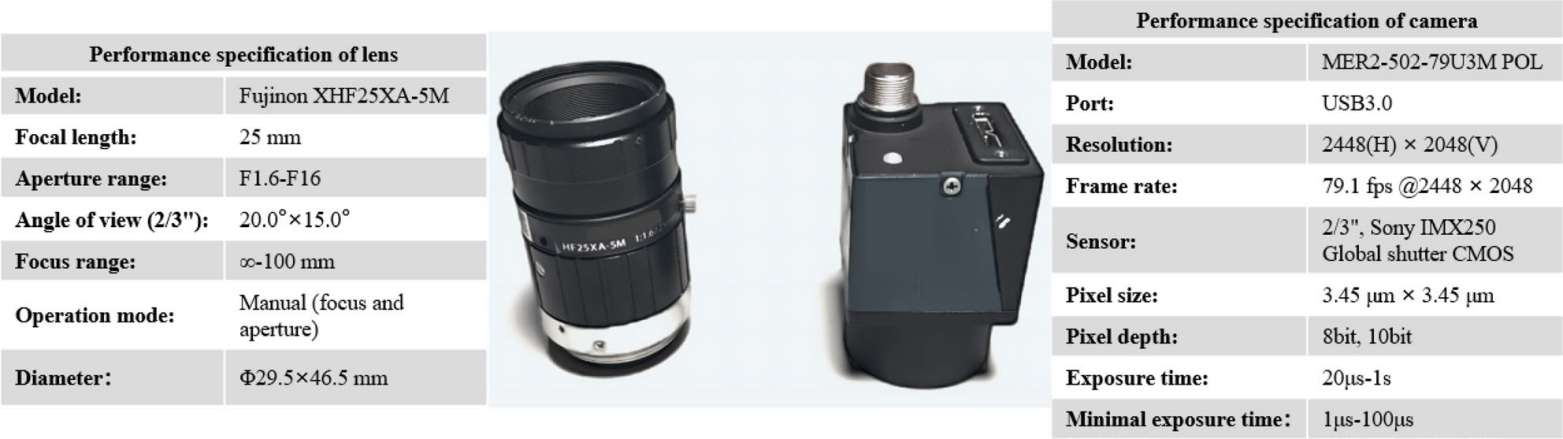
Physical picture and performance specification of the camera and lens.

**Figure 2:**
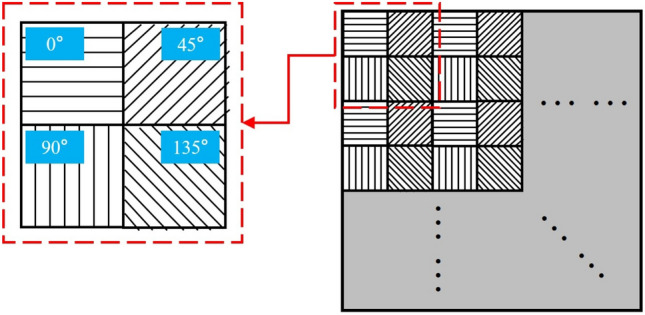
Schematic diagram of the pixelated micro-polarizer array.

The camera for measuring the target polarization characteristics is a division-of-focal-plane (DoFP) camera independently developed by Daheng Optics. The lens is XHF25XA-5 M by Fujinon, a prime lens with a focal length of 25 mm. The physical picture and performance specification of the camera and lens are shown in Fig. 1. The schematic diagram of the pixelated micro-polarizer array is shown in Fig. 2. Four pixels in different directions form a superpixel, and the camera can directly obtain the light intensity images in the four directions. The linear Stokes vector images of the reflected light can be calculated by Eq. (1).

1 $$ {\mathbf{S}} = \left[ {\begin{array}{*{20}c} {S_{0} } \\ {S_{1} } \\ {S_{2} } \\ \end{array} } \right]{ = }\left[ {\begin{array}{*{20}c} {I_{{0^{ \circ } }} + I_{{90^{ \circ } }} } \\ {I_{{0^{ \circ } }} - I_{{90^{ \circ } }} } \\ {I_{{45^{ \circ } }} - I_{{135^{ \circ } }} } \\ \end{array} } \right] $$

where $I_{{0^{ \circ } }}$, $I_{{45^{ \circ } }}$, $I_{{90^{ \circ } }}$ and $I_{{135^{ \circ } }}$ are the light intensity images in four different directions respectively. According to the definition of the degree of linear polarization (DoLP), the DoLP image can be calculated by Eq. (2).

2 $$ {\text{DoLP}} = \frac{{\sqrt {S_{1}^{2} + S_{2}^{2} } }}{{S_{0} }} $$

where $S_{0}$ represents the total intensity of the reflected light; $S_{1}$ and $S_{2}$ represent the magnitude and orientation of the semi-major axis of the polarization ellipse. The circularity of the polarization ellipse, which is necessary for calculating the degree of polarization (DoP), is usually negligible after reflection^10^. In addition to the limitations of the DoFP camera, the polarization characteristics below is limited to DoLP.

**Figure 3:**
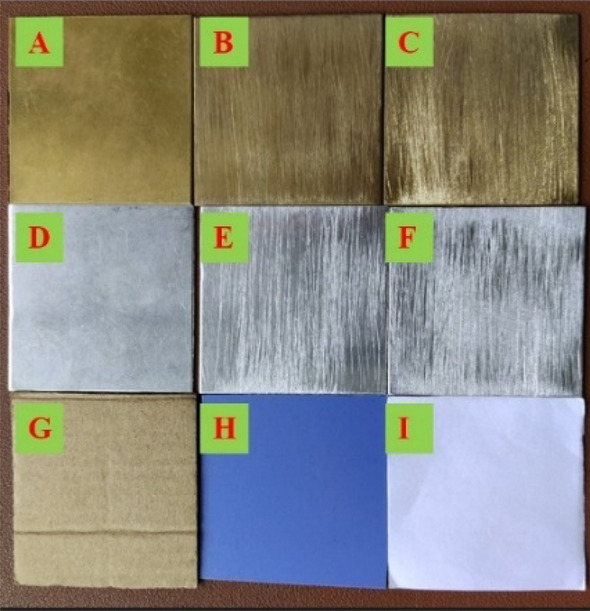
Experimental samples.

The experimental samples include 3 brass plates with different roughness (A–C), 3 aluminum plates with different roughness (D–F), cardboard (G), plastic (H) and white paper (I), as shown in Fig. 3. The metallic material samples A–F are chosen to be the same samples in references^23–25^ and the non-metallic material samples G–I are common materials to explore the effect of material on polarization characteristics. The different roughness is produced by sandpaper with different mesh numbers.

The surface roughness of the experimental samples was measured under the Ra option of TR100 surface roughness tester, where Ra means the arithmetic average of the absolute distance between each point on the measured profile and the midline of the profile, within 0.25 mm sampling length. Since the surface roughness was not uniform, 10 different positions were randomly selected on the surface for measurement, and the mean value was regarded as the surface roughness. The measurement results are shown in Table 1.

**Table 1:** Measurement results of sample surface roughness.

Sample	Measurement results /μm	Mean /μm
A	0.16; 0.11; 0.25; 0.23; 0.16; 0.18; 0.21; 0.23; 0.19; 0.18	0.19
B	1.72; 1.00; 1.81; 1.93; 1.97; 1.78; 1.93; 1.88; 1.75; 1.82	1.56
C	2.58; 2.71; 3.02; 2.55; 2.56; 2.66; 2.78; 2.85; 2.66; 2.78	2.71
D	0.34; 0.29; 0.34; 0.34; 0.32; 0.34; 0.30; 0.32; 0.30; 0.28	0.32
E	1.26; 1.52; 2.00; 1.65; 1.84; 1.76; 1.95; 1.85; 1.75; 1.95	1.75
F	3.62; 3.38; 4.44; 3.75; 3.03; 3.43; 4.69; 4.85; 3.45; 3.65	3.52
G	3.14; 2.96; 3.02; 2.89; 3.34; 3.66; 3.96; 3.27; 2.90; 3.21	3.24
H	1.12; 1.04; 1.78; 1.53; 1.53; 2.05; 1.36; 1.30; 1.23; 1.23	1.42
I	2.30; 2.02; 2.17; 2.07; 2.50; 2.30; 2.39; 2.32; 2.40; 2.21	2.17

### Experimental condition settings

**Figure 4:**
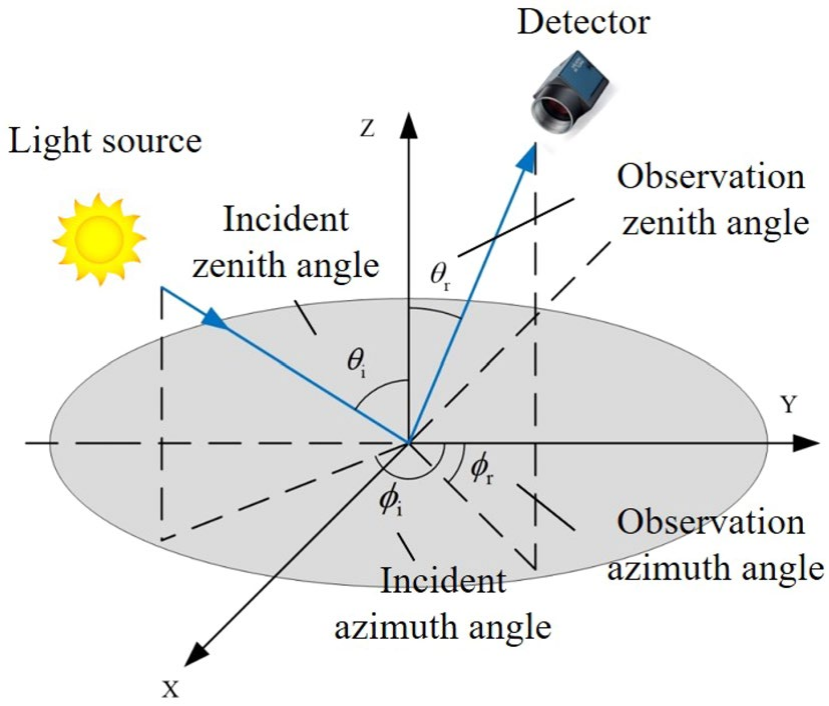
Schematic diagram of measurement.

The schematic diagram of measurement is shown in Fig. 4, where, the light source is the sun; the detector is the DoFP camera; $\theta_{{\text{i}}}$ is the incident zenith angle; $\theta_{{\text{r}}}$ is the observation zenith angle; $\phi_{{\text{i}}}$ is the incident azimuth angle; $\phi_{{\text{r}}}$ is the observation azimuth angle.

The experimental condition settings are shown in Table 2.

**Table 2:** Experimental condition settings.

Conditions	Settings	Conditions	Settings
Location	Xi’an Hongqing	Environment	Outdoor
Longitude	108.501°E	Latitude	29.735°N
Weather	Sunny	Local time	09:00–10:00
Incident zenith angle	45°	Incident azimuth angle	0°
Object distance	1.7 m	Aperture range	F8 (fixed)

**Figure 5:**
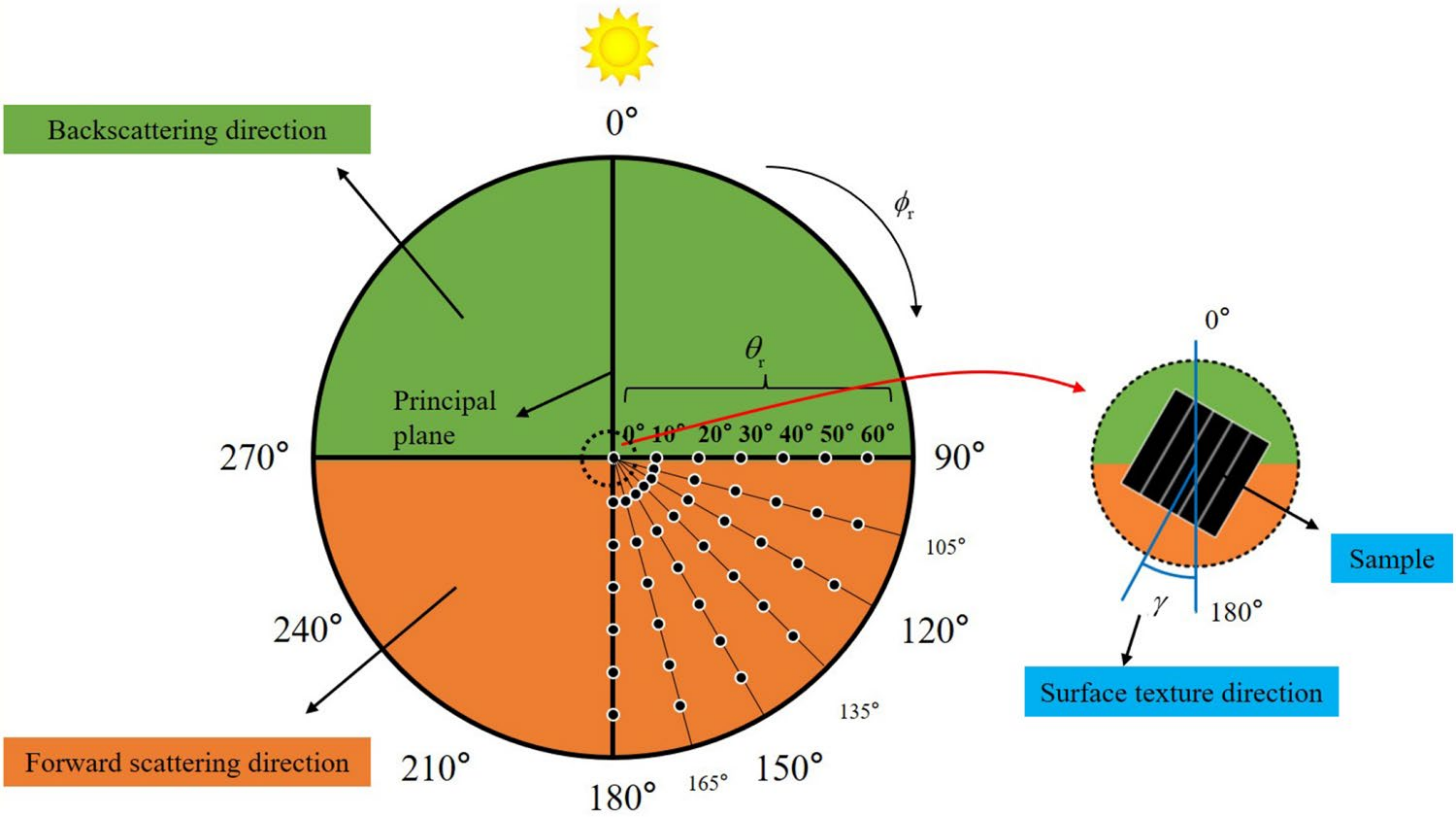
Schematic diagram of measuring positions and surface texture direction.

During the experiment, though the incident zenith angle has changed from 45° to 48°, the influence is the influence of the change is small and negligible, so the incident zenith angle is uniformly considered to be 45°. In the measurement process, because the shadow of the detector will affect the results when measuring in the backscattering direction, the experiment mainly focuses on the forward scattering direction. To reduce the measurement time to prevent the solar incident zenith angle from changing too much, the polarization characteristics are assumed to be symmetrical with respect to the principal plane. The samples are measured when the observation azimuth angle is 90–180°, with an interval of 15°, and the observation zenith angle is 0–60°, with an interval of 10°. The total measuring positions are 29, which are shown in Fig. 5. The circumferential direction refers to the observation azimuth angle change, the radial direction refers to the observation zenith angle change, and the solid points are the actual measuring positions.

When making experimental samples, the surface roughness of samples B, C, E and F were generated in the same direction to produce surface rough texture structure with a strong direction. To analyze the influence of the surface texture direction on the polarization characteristics of the samples, during the experiment, the whole experimental samples were rotated 45° and 90° around the geometric center and repeated the above measurement process. The surface texture direction is defined as the angle between the texture direction of the samples and the principal plane. Under three rotations, the surface texture direction $\gamma$ was 0°, 45°, and 90°, respectively.

## Results and analysis

**Figure 6:**
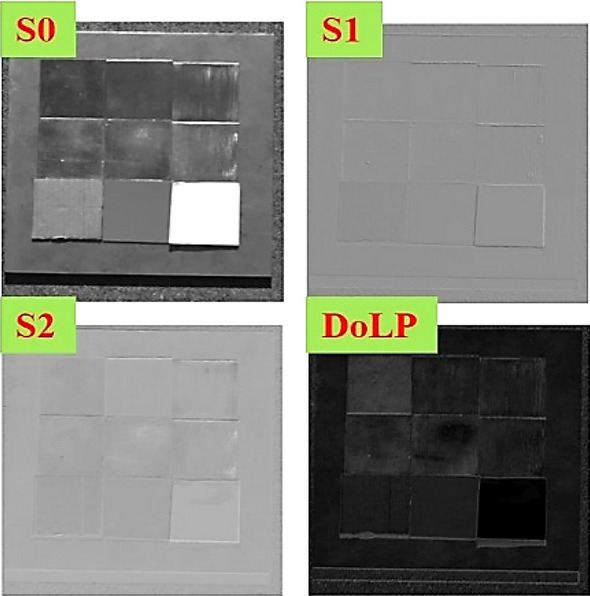
Linear Stokes vector and DoLP images of the samples.

The measurement adopts the polarization imaging method and each image taken contains the nine samples from the experiment. So, the DoLP of a sample can be obtained through calculating the average gray value of pixels belonging to the sample in the image. The linear Stokes vector and DoLP images obtained from the measurement are shown in Fig. 6.

Based on the above method, DoLP of 9 samples at each measuring position are calculated, and influence factors of target polarization characteristics are analyzed respectively on the principal plane and in the forward scattering space.

### Polarization characteristics on the principal plane

**Figure 7:**
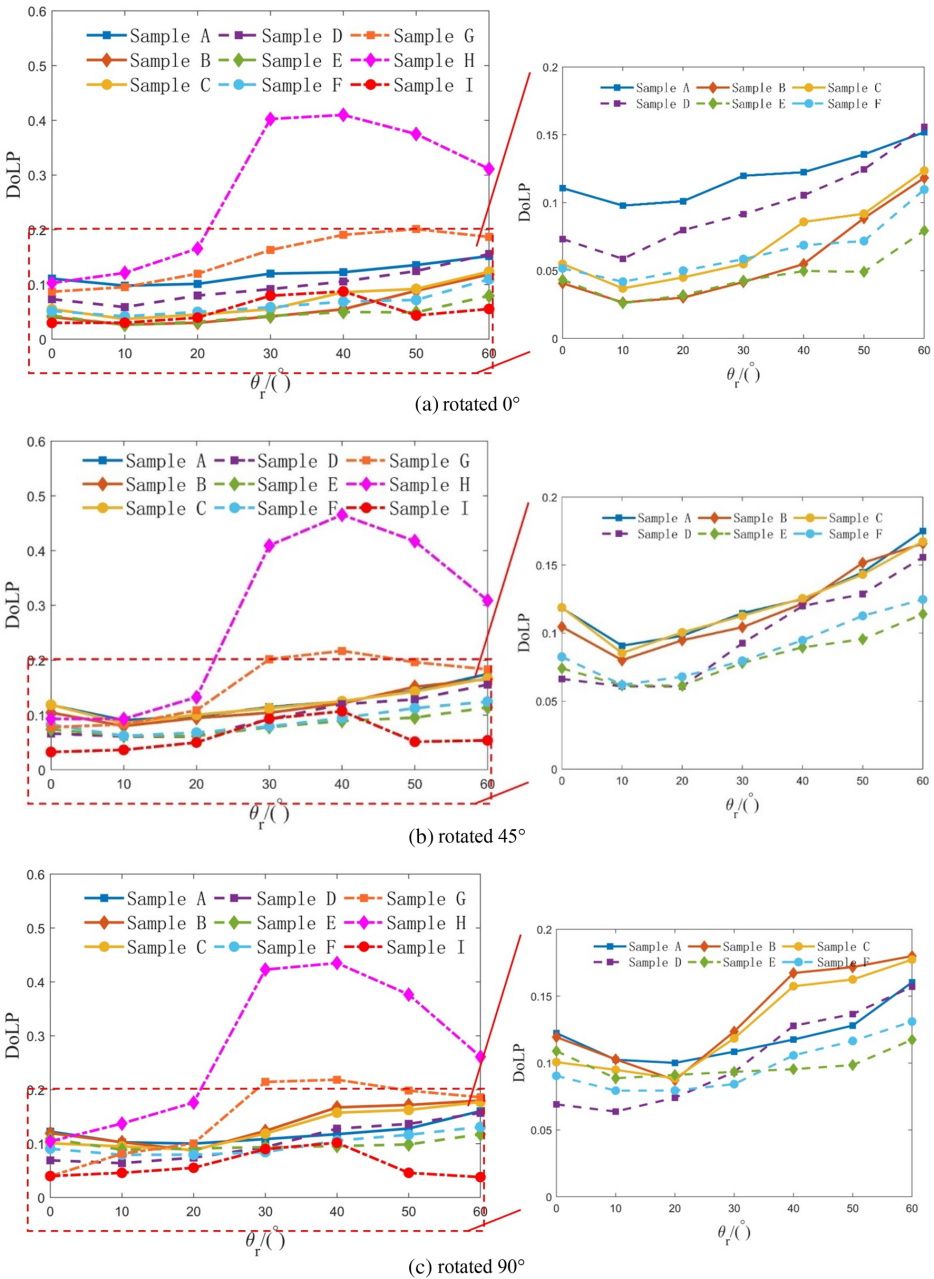
DoLP of samples A-I at different observation zenith angles on the principal plane, when samples are (**a**) rotated 0°, (**b**) rotated 45°, and (**c**) rotated 90°.

The polarization information is mainly generated by specular reflection, which will make the polarization directions consistent to produce a large degree of polarization^22^. Thus, the DoLP on the principal plane with the strongest specular reflection is selected to analyze the influence factors of the target polarization characteristics. Under three rotations, the DoLP of samples A-I at different observation zenith angles on the principal plane are shown in Fig. 7.

As shown in Fig. 7, the DoLP of different materials varies not only in value but also has a different variation trend with the observation zenith angle. As for non-metallic material samples G–I, since there is no obvious surface texture direction, the DoLP value and the trend of variation with the observation zenith angle do not change significantly under three rotations. The DoLP ranges of sample G–I are 0–0.5, 0–0.2, and 0–0.1, respectively. As the observation zenith angle increases from 0° to 60°, the DoLP of the three increases first and then decreases, and reaches its maximum value near the specular reflection position (30–40°), where the specular reflection is the strongest, and the polarization information generated is also the most.

As for metallic material samples A-F, the DoLP ranges are all 0.05–0.2. As the observation zenith angle increases from 0° to 60°, the DoLP reaches the minimum value when the observation zenith angle is 10–20°, and then increases with the increase of the observation zenith angle. In particular, for the brass plate sample A and the aluminum plate sample D with relatively smooth surface and no obvious texture direction, the DoLP value and the trend of variation with the observation zenith angle do not change significantly under three rotations and the DoLP of sample A is larger than that of sample D, which is the same as the measurement results in reference^23,24^. For the brass plate samples B–C and the aluminum plate samples E–F with relatively rough surfaces and obvious texture direction, the DoLP value and the trend of variation with the observation zenith angle change obviously under three rotations. When samples are rotated 0°, the DoLP of smooth samples A and D is larger than that of rough samples B–C and E–F respectively, but similar when rotated 45° and even smaller at some observation zenith angles when rotated 90°. This does not mean that it is contrary to the conclusion in reference^25^ that the surface roughness is inversely proportional to DoLP, but that when the rough surface has an obvious texture direction, the surface texture direction of the target surface should be taken into account.

The above analysis shows that the polarization characteristics of the target are related to the target material, observation zenith angle, surface roughness, and texture direction.

### Polarization characteristics in the forward scattering space

**Figure 8:**
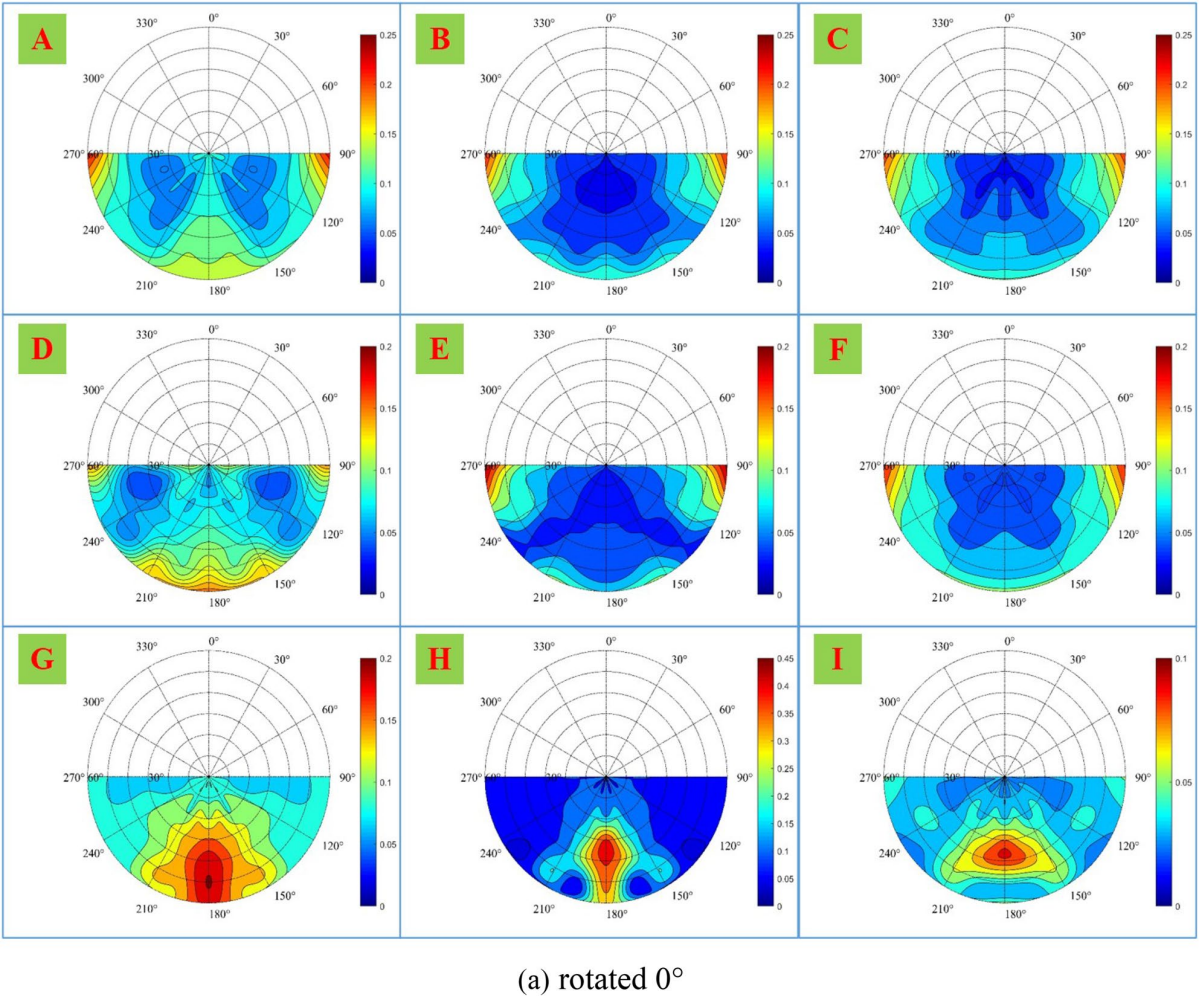

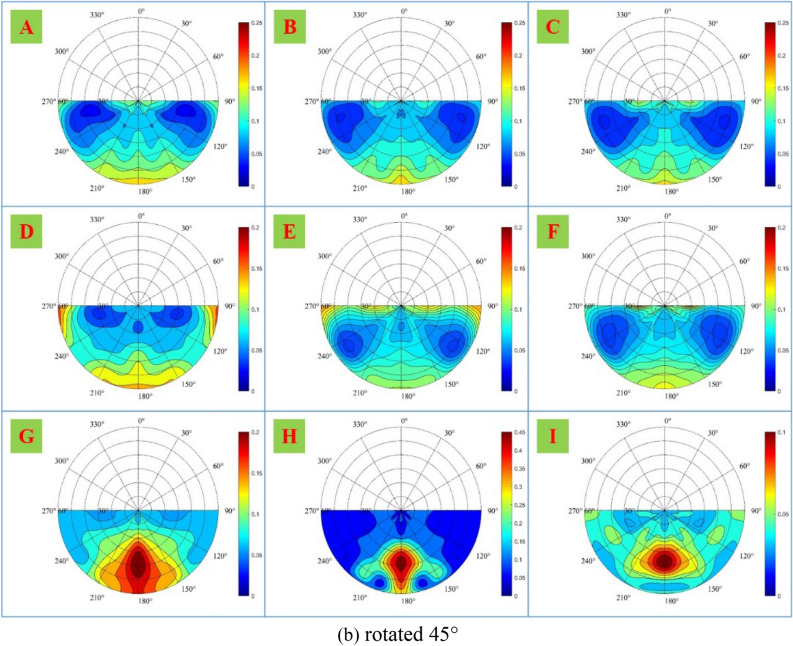

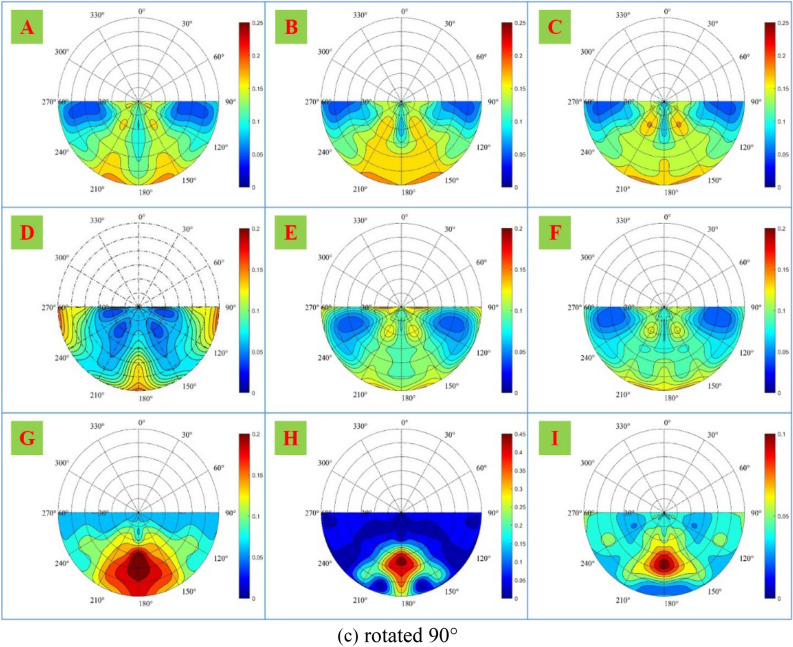
DoLP of samples A-I in the forward scattering space, when samples are (**a**) rotated 0°, (**b**) rotated 45°, and (**c**) rotated 90°.

To further analyze the spatial distribution of the polarization characteristics, the DoLP of samples A-I in the forward scattering space is shown in Fig. 8.

As shown in Fig. 8, the DoLP distribution of different materials in the forward scattering space is different. As for non-metallic material samples G–I, since there is no obvious surface texture direction, the DoLP distribution of each sample in the forward scattering space is similar under three rotations. Since the three samples are with strong forward scattering, the polarization information is mainly concentrated near the principal plane and reaches its maximum at the specular reflection position.

As for metallic material samples A–F, theoretically, the spatial distribution of polarization characteristics of samples A and D without obvious surface texture direction should not change significantly under three rotations. However, from the experimental results, the change of sample D is small, while the change of sample A is large, which may be due to the error caused by the change of relative position during rotation. For samples B–C and E–F, due to the obvious surface texture direction, the spatial distribution of polarization characteristics changes significantly under three rotations. When samples are rotated 0°, the polarization characteristics are not concentrated on the principal plane, but on the observation azimuth angle of 90° and 270°; when rotated 45°, the polarization characteristics begin to concentrate on the principal plane and decrease on the observation azimuth angle of 90° and 270°; and when rotated 90°, the polarization characteristics are mainly concentrated on the principal plane. The analysis above shows the spatial distribution of the polarization characteristics is related to the surface texture direction.

### Influence of surface texture direction on polarization characteristics

**Figure 9:**
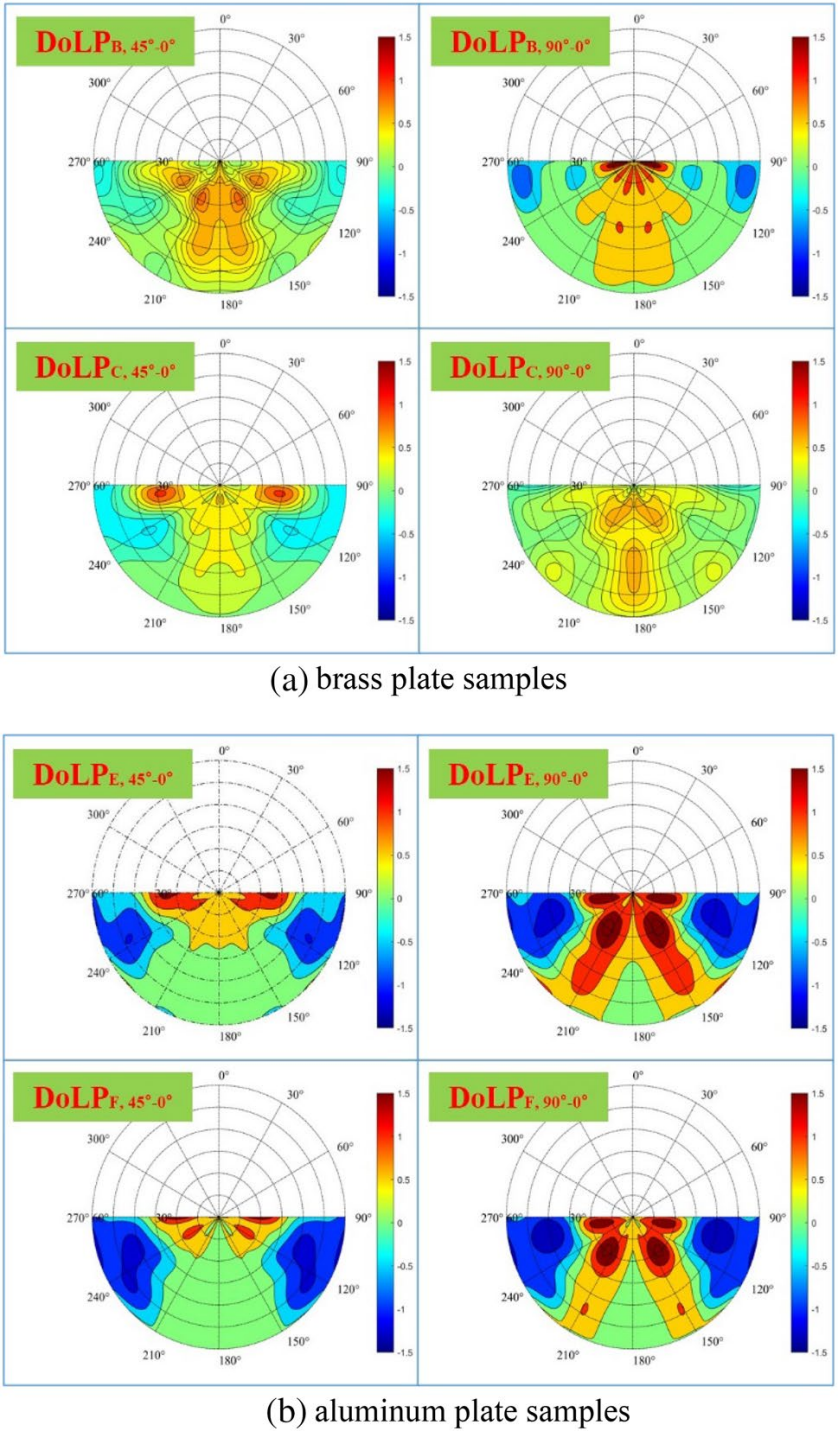
The influence of surface texture direction on the spatial distribution of polarization characteristics of (**a**) brass plate samples and (**b**) aluminum plate samples.

To further analyze the specific influence of surface texture direction on the spatial distribution of target polarization characteristics and eliminate the error caused by the relative position change during rotation, samples A and D of each group are used as the control group and the DoLP of samples B–C and E–F are normalized by dividing the DoLP of samples A and D of this group, respectively. Finally, the DoLP of normalized B–C and E–F samples rotated 45° and 90° is compared with the corresponding sample rotated 0°, and the change of the spatial distribution of normalized polarization characteristics before and after rotation can be obtained by Eq. (3).

3 $$ {\text{DoLP}}_{{{\text{n}},{\text{j}}^{ \circ } { - 0}^{ \circ } }}  = \frac{{{\text{DoLP}}_{{{\text{n}},{\text{j}}^{ \circ } }} }}{{{\text{DoLP}}_{{{\text{m}},{\text{j}}^{ \circ } }} }} - \frac{{{\text{DoLP}}_{{{\text{n}},{0}^{ \circ } }} }}{{{\text{DoLP}}_{{{\text{m}},{0}^{ \circ } }} }} $$

where ${\text{DoLP}}_{{{\text{n}},{\text{j}}^{ \circ } }}$ is the DoLP of sample n $\left( {\text{n}} = {\text{B,C,E,F}} \right)$, when rotated ${\text{j}}^{ \circ }$ $\left( {\text{j}} = 45,90 \right)$;${\text{DoLP}}_{{{\text{m}},{\text{j}}^{ \circ } }}$ is the DoLP of control sample m $\left( {\text{m}} = {\text{A,D}} \right)$, when rotated ${\text{j}}^{ \circ }$ $\left( {\text{j}} = 45,90 \right)$; ${\text{DoLP}}_{{{\text{n}},{\text{j}}^{ \circ } { - 0}^{ \circ } }}$ is the normalized DoLP difference between the sample n when rotated ${\text{j}}^{ \circ }$ and 0°. Through the calculation above, the specific influence of surface texture direction on the spatial distribution of target polarization characteristics can be obtained. It is worth mentioning that the normalized DoLP of samples A and D of the control group is 1, and the difference is 0, which means that rotation has no effect on the samples of the control group with relatively smooth surfaces and no obvious texture direction. The results of samples B, C, E, and F are shown in Fig. 9.

As shown in Fig. 9, the surface texture direction has different effects on the spatial distribution of target polarization characteristics of different materials. For the brass plate (samples B and C), the DoLP on and near the main plane increases when rotated 45° and 90°, and the increase amplitude when rotated 90° is greater than that when rotated 45°. For aluminum plates (samples E and F), when rotated 45°, the DoLP increases in the all-azimuth space where the observation zenith angle is 0–20°, which is in the space directly above the sample, while the DoLP of the sample is smaller in the space where the observation zenith angle is in the range of and the observation azimuth angle is in the range of 90–130° and 230–270°; When rotated 90°, the change is similar to the former, but the DoLP near the principal plane increases. The analysis above again shows that the target surface texture direction affects the spatial distribution of the target polarization characteristics, and the greater the angle between the surface texture direction and the principal plane, the more concentrated the target polarization characteristics are in and around the principal plane. The main reason is s that regular grooves are formed on the surface of the sample, when roughness is produced. When the greater the angle between the surface texture direction and the principal plane, the more mirror reflection is generated by the microfacets on the sample surface, so the more concentrated the polarization information generated in the forward scattering space. On the contrary, when the angle is small, the specular reflection is dispersed in other directions, causing the polarization information away from the principal plane.

## Conclusion

In this paper, the polarization characteristics of 3 brass plates with different roughness, 3 aluminum plates with different roughness, cardboard, plastic and white paper, a total of 9 samples are measured in an outdoor environment. Then, the influencing factors of the target polarization characteristics are analyzed respectively in the principal plane and in the forward scattering space. Finally, the influence of the surface texture direction is emphatically discussed by defining the normalized difference function. The results show that the target polarization characteristics are related to the material, surface roughness, detection position, and surface texture direction, where, the surface texture direction affects the spatial distribution of the target polarization characteristics, and the greater the angle between the surface texture direction and the principal plane, the more concentrated the target polarization characteristics are in and around the principal plane.

## Data Availability

The datasets used and analysed during the current study available from the corresponding author on reasonable request.
